# Frequency of ABH secretors and non secretors: A cross sectional study in Karachi

**DOI:** 10.12669/pjms.301.4194

**Published:** 2014

**Authors:** Muhammad Saboor, Aman Ullah, Khansa Qamar, Awal Mir, Moinuddin

**Affiliations:** 1 Dr. Muhammad Saboor, PhD, Baqai Institute of Hematology, Baqai Medical University, Karachi, Pakistan.; 2 Aman Ullah, M.Sc (Hematology), Baqai Institute of Hematology, Baqai Medical University, Karachi, Pakistan.; 3 Khansa Qamar, M.Sc (Hematology), Baqai Institute of Hematology, Baqai Medical University, Karachi, Pakistan.; 4 Awal Mir, M.Sc (Hematology), Baqai Institute of Hematology, Baqai Medical University, Karachi, Pakistan.; 5 Dr. Moinuddin, FRCP (C), FRCP (E), Baqai Institute of Hematology, Baqai Medical University, Karachi, Pakistan.

**Keywords:** ABO group, ABH, Secretors, Non-secretors

## Abstract

***Objective: ***ABO blood group and secretor status is valuable in relation to some diseases in clinical and forensic medicine. Across the globe there are geographic and racial differences in the frequency of secretors and non-secretors. Aim of this study was to evaluate the status of ABH blood group secretors and non-secretors in Karachi (Pakistan).

***Methods:*** Blood and saliva samples were randomly collected from one hundred and one (n=101) healthy adult students (76 male, 25 female) ranging in age from 15 to 40 years. Their ABO and Rhesus blood groups were determined by conventional methods, and their secretor status was studied by hemagglutination inhibition method of saliva.

***Results:*** Results showed that 64.4% of the study population were ABH blood group secretors while 35.6% were non-secretors. Frequencies of the secretor status among various ABO blood groups were 71.4% in group A, 79.5% in group B, 45.5% in group AB, and 61.5% in group O.

***Conclusion:*** Frequency of ABH secretor is high (64.4%). Blood group B has the highest secretor (79.5%) frequency while Blood group AB has the lowest (45.5%).

## INTRODUCTION

An Austrian Scientist Karl Landsteiner discovered the ABH blood group system in 1901.^1^ In 1926 it was found that A and B antigens were not only present on red cells but they were also present in soluble form in saliva. In 1930, Putkonen noted that a person could be either secretor or non-secretor with respect to his genetic ability to secrete ABH blood group substances in secretions.^2^ It is now known that ABH blood group antigens (A, B and H) are found on red blood cells, lymphocytes, platelets, tissue cells, body fluids (except CSF) and in secretions.^3^^,^^4^ Basic differences between secretors and non-secretors are qualitative and quantitative components of their saliva, mucus and other body secretions.^5^

ABH secretions are controlled by fucosyletransferase 2 (FUT2) secretor gene located on the short arm of chromosome number 19 in the form of two alleles denoted as “Se” and “se”. The Se is dominant while se is recessive (or amorphic) in their pattern of inheritance. SeSe and Sese produce a dominant secretor phenotype while sese produces a recessive non-secretor phenotype. The FUT2 gene encodes for enzyme glycosyletransferase (α-2-L-Fucosyl transferase) which become active in goblet cells, and mucus glands of gastrointestinal tract (saliva, bile, gastric juice, mucus), urogenital tract (seminal fluid, vaginal secretions and urine) and respiratory tract as well as in the sweat, tears, milk and amniotic fluid.^6^

ABH secretors secrete ABH substances according to their blood groups. “O” blood group secretes only H substance, A blood group secretes A and H substances while B blood group secretes B and H substances in the fluids.^7^^,^^8^

Saliva of ABH secretors contains additional carbohydrate compounds in themucin that aggregate certain bacteria and decrease their activity. Non-secretors have high incidence of diseases of mouth, esophageal cancer, epithelial dysplasia as compared to secretors.^9^ ABH soluble substances in intestinal secretions increase the brush border hydrolase enzyme activity that has significant effects on bacteria and lectin adherence to the microvilli of the intestine. Soluble ABH substances in intestine prevent the attachment of *H. pylore* to the gut wall and decrease the incidence of *H.pylore* infections.^10^

ABH secretor status is also important for the growth of normal flora which is responsible for the normal functioning of gastrointestinal tract. It has been found that some bacteria in the GI tract produce ABH degrading enzymes and use ABH substances for constant food supply. Bacteria capable of degrading blood group “B” antigen produce enzymes that allow them to detach the terminal alpha-D-galactose and use these sugars for food. Blood group “A” degrading bacteria have similar capability with respect to N-acetylgalactoseamine. On the other hand absence of ABH substances in intestinal and urogenital tract secretions allow microorganisms adherence and increase the risk of duodenal ulcer, celiac disease, urinary tract infections and persistent candida infections.^11^

Frequency of secretors and non-secretors of ABO blood group system has not been determined yet in Pakistan. Aim of this study was to evaluate the prevalence of ABH blood group secretors and non-secretors in Karachi (Pakistan).

## METHODS

It is a cross sectional study conducted for the evaluation of ABH secretor and non- secretor status in the Karachi (Pakistani) population. One hundred and one healthy adult students (76 male, 25 female) between 15 to 40 years of age were randomly selected for this study. Written informed consent was obtained from all individuals. This study was approved by the ethical committee of Baqai Medical University, Karachi. Ten ml saliva was collected in sterilized disposable plastic containers from all individuals. Five ml (2 ml in EDTA tube while 3 ml in un-anticoagulated tube) blood was collected form all individuals aseptically for cell and serum grouping.

***ABO and Rh blood grouping procedure:***

***Preparation of 3% red cells suspension:*** Two ml of blood collected in an EDTA anti-coagulated tube was centrifuged at low speed (1500 rpm) for 3 minutes. Plasma was transferred into another tube and used in serum grouping. Tube containing cells was filled with normal saline. The tube was well mixed and re-centrifuged at 1500 rpm for 3 minutes. Washing procedure was repeated twice. During last washing, the tube was centrifuged at high speed (3000rpm) for 3 minutes and the supernatant was discarded. Transparent supernatant and appearance of cell trailing line indicates proper cell washing.

***I: Cell grouping:*** Three clean glass test tubes were labeled as tube A for anti A, tube B for anti B and tube D for anti D. One drop of antiserum was added as Anti A sera to tube A, anti B sera to tube B and anti D sera to tube D. One drop of 3% red cell suspension was added to tube A, tube B and tube D. All contents were well mixed. All tubes were centrifuged immediately by balancing at 3400 rpm for 15 seconds. All tubes were taken out gently and tube was examined individually for agglutination.

***II: Serum grouping: ***Serum was separated from un-anticoagulated tube after centrifugation for 5 minutes at 3000rmp. Three clean glass test tubes were labeled as tube “a” for A cells, tube “b” for B cells and “o” for O cells. Two drops of individual’s serum was added to each tube. One drop of known 3% red cells suspension of A, B and O cells was added to tube “a”, “b” and “o”. All tubes contents were well mixed and immediate spin for 15 seconds. All tubes were taken out gently and tube was examined individually for agglutination.

All samples were tested within 6 hours, using fresh red cells suspension of the same individual. Collection and processing of saliva: Ten ml saliva was collected from all individuals in a sterilized disposable plastic container and transferred from the container into a sterilized disposable glass tube. Tube containing saliva was centrifuged at 2000 rpm for 10 minutes. Supernatant was transferred into another glass tube. Tube containing the saliva supernatant was placed in a water bath at 56^o^C for 30 minutes for inactivating salivary amylase. Sample tube was cooled and re-centrifuged for 10 minutes at 2000 rpm. Supernatant was transferred into another disposable test tube and used for the hemagglutination inhibition or neutralization test.

***Principle of hemagglutination inhibition test:*** ABH substances present in soluble form in fluids (e.g., saliva) neutralize their corresponding antibodies. The antibodies will no longer be able to agglutinate red cells possessing the same antigens.^11^^,^^12^

***Hemagglutination inhibition test procedure:***

***Preparation of blood group antisera doubling dilutions: ***Ten test tubes were placed in a rack labeled according to the titer i.e. 1:2 (tube1), 1:4 (Tube 2), up to 1:1024 (tube10). Two hundred micro liter saline was added to all tubes. To the tube 1, 200-µl antisera was added and mixed well. Two hundred µl tube1 dilutions was transferred to tube2 and mixed well. The same procedure was repeated from tube 2 to tube10. Two hundred µl diluted antisera was discarded from tube10. Each tube contained equal amount of antisera and saline. This doubling dilution solution was used in hem agglutination inhibition test.

Three rows of 10 test tubes were placed in a rack and labeled as; I: Doubling dilution (D/D) tubes II: Control tubes (Titration tubes) III: Test tubes (Inhibition tubes).

Doubling dilution of group specific antisera was prepared in row I. Each tube was labeled according to the titer i.e. 1:2 (tube1), 1:4 (tube2) up to 1:1024 (tube10). One hundred µl blood group specific antisera of each dilution was transferred to the correspondingly labeled tubes in the rows II and III as shown in Table-I. One hundred µl of saline was added to each tube in row II. One hundred µl of saliva was added to each tube in row III. All tubes of rows II and III were capped and mixed well. These tubes were incubated at 37°C for 30 minutes in a water bath with periodic shaking. After incubation 100 µl of 3% group specific red cells suspensions was added to each tube in rows II and III and was mixed well. All tubes were incubated at 37°C for 30 minutes. All tubes were observed for agglutination macroscopically under good light source.

***Interpretation of results:***

- Positive secretor status:

Macroscopic agglutination at higher titer (1:128) in control (row II) and lower titer (1:16) in test (row III) indicated the presence of blood group substances in the test saliva.

- Negative secretor status:

No difference in the titer at which visible agglutination in both rows implied the absence of blood group specific substances in the saliva to neutralize the antiserum.^13^

**Table-I:** Setting of the tubes as per study protocol

*Table*	*Rows*		
	*I*	*II*	*III*
	*D/D* *Saline + Antisera*	*Controls* *Saline + Each dilution*	*Test* *Saliva + Each Dilution*
1 (1:2)	+	+	+
2 (1:4)	+	+	+
3 (1:8)	+	+	+
4 (1:16)	+	+	+
5 (1:32)	+	+	+
6 (1:64)	+	+	+
7 (1:128)	+	+	+
8 (1:256)	+	+	+
9 (1:512)	+	+	+
10 (1:1024)	+	+	+

Antisera=100 µl, Saline=100 µl, Saliva=100 µl, Each dilution=100 µl, D/D = Double dilution

**Statistical analysis: **Statistical package for social sciences (SPSS) version 17 was used for data analysis. Descriptive statistics was used for the calculation of frequency and percentages. 

## RESULTS

Total number of individuals enrolled in this study was 101 (76 male and 25 female) and their age ranged between 18 - 40 years with an average of 23 years. Of the studied population, 64.4% individuals were found to be secretors and 35.6% were non-secretors. There was no gender difference on the frequency of secretor and non-secretor status as shown in Table-II.

**Table-II:** Distribution and comparison of secretor status in the studied population

Gender	Secretor		Non-secretor	
	N	Frequency (%)	N	Frequency (%)
Male	49	64.5	27	35.5
Female	16	64	09	36

In Rh (D) positive individuals 63.6% were secretors and 36.4% were non-secretors while Rh (D) negative individual were 76.9% secretors and 23.1% were non-secretors as shown in Table-III. Distribution of ABO blood groups and frequency of secretor status in A, B, AB and O blood groups in the study are shown separately in Table-IV.

**Table-III:** Incidence of secretors in male and females in Rh blood group system

Secretor status	Rh positive		Rh negative	
	Male	Female	Male	Female
Secretors	42	14	08	02
Non-secretors	23	09	03	00
Total	65	23	11	02

**Table-IV:** Frequency of secretor and non secretor status in A, B, AB, O and Rh blood groups

Variables	Blood Groups					
	A	B	AB	O	Rh Positive	Rh Negative
Secretors (n)	20	25	05	16	56	09
Frequency (%)	71.4	79.5	45.5	61.5	63.6	69.2
Non secretors (n)	08	11	06	10	32	04
Frequency (%)	28.6	20.5	54.5	38.5	36.4	30.7

## DISCUSSION

In this study the frequency of ABH secretors is 64.4% and for non-secretors was 35.6%. Prevalence of different ABO and Rh D blood groups was 27.72% of group ‘A’; 35.64% ‘B’; 10.89% ‘AB’ and ‘O’ 25.75% respectively. 87.13% o o o f the subjects were Rh positive while 12.87% were Rh negative. Frequency of ABO blood group in this study is similar to other studies conducted in Pakistan.^14^ There is no previous available data on the frequency of ABO and Rh secretor status in Pakistan. There was no gender variation in the frequency of secretor and non-secretor in the present study.

Frequency of ABH secretor status in the world population is about 80% secretors and 20% non-secretors with some geographic and racial differences.^15^ Percentage of secretors and non-secretors in Negroes correlates well with that our study.^16^ Findings of this study are at variance with that conducted in American, German, Iraqi, Japanese and Bangladeshi population.^15^^-^^17^

In this study, blood group B has the highest secretor (79.5%) frequency while Blood group AB has the lowest secretor (45.5%) level. Blood group A and O have 71.4% and 61.5% secretor positivity respectively.

ABO blood groups and secretors status has some relevance to certain diseases and clinical and forensic medicine. Malignancies like leukemias and non-Hodgkin lymphomas alter the red cells antigens and show weaker reactivity during cell grouping. In such patients saliva studies may help to confirm the patient actual blood group if the individual is a secretor. There are certain advantages of having ABH substances in saliva and other secretions. Also there are also certain diseases which show higher incidence association with non-secretors.^15^^,^^17^

ABH substances in saliva are most significant for smooth surface area of the teeth. In all blood groups the incidence of dental cavities is lower in ABH secretors than for non-secretors.^5^

Secretor status and ABH genetics affect up to 60% of the vWf and FVIII concentration in plasma. Non-secretors individuals have shorter bleeding time due to higher plasma concentration of vWf and factor VIII level that has a greater risk for thrombosis and heart disease.^18^ It was also found that blood group “O” secretor have the lowest concentration of vWf Ag and factor VIII Ag.^19^

ABH non-secretors also have a higher prevalence of certain auto-im-- mune diseases like ankylosing spondylitis, reactive arthritis, psoriatic arthropathy, Sjogren’s syndrome, multiple sclerosis and Grave’s disease.^13^

## Authors Contribution:

Muhammad Saboor carried out the research work, statistical analysis and writing of manuscript. 

Dr. Moinuddin supervised, reviewed and approved the final manuscript.

Khansa Qamar and Aman Ullah and Awal Mir worked as co-researchers.

## Notes

***Grant Support & Financial Disclosures: ***Baqai Institute of Hematology, Baqai Medical University.
